# Analysis of Factors Affecting Postoperative Opioid Requirement in Adult Patients Undergoing Minimally Invasive Repair of Pectus Excavatum

**DOI:** 10.3390/jcm15031023

**Published:** 2026-01-27

**Authors:** Minju Kim, Saewon Park, Seung Keun Yoon, Wonjung Hwang

**Affiliations:** 1 Department of Anesthesiology and Pain Medicine, Seoul St. Mary’s Hospital, College of Medicine, The Catholic University of Korea, Seoul 06591, Republic of Korea; jkkpsk@gmail.com (M.K.); cewonp215@naver.com (S.P.); 2 Department of Thoracic and Cardiovascular Surgery, Seoul St. Mary’s Hospital, College of Medicine, The Catholic University of Korea, Seoul 06591, Republic of Korea

**Keywords:** analgesia, analgesics, opioid, pain, postoperative, pectus excavatum

## Abstract

**Background/Objectives**: Minimally invasive repair of pectus excavatum (MIRPE) is an established surgical treatment for adult pectus excavatum (PE). Compared with pediatric patients, adults generally have a more rigid chest wall and greater costal cartilage ossification, often resulting in more severe postoperative pain. However, most previous studies have focused on pediatric patients with PE and on evaluating effective analgesic methods. This study aimed to investigate perioperative factors associated with postoperative opioid requirements and pain intensity in adult patients undergoing MIRPE. **Methods**: This study was a single-center retrospective study of adult PE patients who underwent MIRPE between March 2011 and January 2023. The primary outcome was total opioid consumption during the first 24 postoperative hours. Secondary outcomes included opioid and rescue analgesic use within 0–6, 6–24, and 24–48 h, as well as pain intensity during each interval. Multivariable linear regression analysis was performed to identify factors associated with postoperative opioid consumption. **Results**: A total of 382 patients were analyzed. Pain intensity peaked within the first 6 postoperative hours, decreased during the 6–24 h and increased during 24–48 h period. Higher BMI and placement of more than three bars were independently associated with greater opioid consumption during the first 6 h (*p* < 0.001). Within 24 and 48 h, male sex, longer operation time and higher BMI were independently associated with opioid consumption (*p* < 0.001). During 6–24 and 24–48 h period, VAS severity was significantly higher in male patients and those with longer operation times. **Conclusions**: Male sex, higher BMI, prolonged operation time, and multiple bar insertion may contribute to greater postoperative opioid requirements during the early postoperative phase in adult patients undergoing MIRPE.

## 1. Introduction

Pectus excavatum (PE) is the most common congenital deformity of the anterior chest wall, characterized by posterior depression of the sternum and adjacent costal cartilages. Although the condition is usually diagnosed in childhood or adolescence, many patients present for surgical correction in adulthood due to persistent cosmetic and functional concerns [1,2]. Among available techniques, the minimally invasive repair of pectus excavatum (MIRPE) has become a commonly performed procedure that elevates the depressed sternum using one or more metal bars inserted beneath it [3,4]. Although MIRPE offers less tissue disruption and better cosmetic outcomes than open repair, postoperative pain remains a major clinical concern—particularly in adults whose increased mature chest wall rigidity and costal cartilage ossification exacerbate tissue stress and require greater intercostal nerve traction pressure during bar rotation, contributing to more postoperative pain compared to children and adolescents [5,6].

Although the severity of postoperative pain after MIRPE in adult patients has been well recognized, the intensity of postoperative pain and the amount of opioid required for adequate analgesia during this immediate postoperative period vary widely among individuals. Understanding the factors that contribute to this variability is essential for optimizing individualized postoperative pain management. However, the perioperative factors associated with increased opioid requirements and pain severity during this period remain insufficiently defined in adult patients undergoing MIRPE. Most prior studies have focused on pediatric or adolescent populations or have primarily evaluated analgesic techniques rather than patient- or surgery-related determinants of pain. Consequently, data specifically addressing factors associated with postoperative pain and opioid consumption in adult MIRPE remains limited and inconsistent.

This study aimed to investigate perioperative factors associated with postoperative opioid requirements and pain intensity during the early postoperative phase in adult patients undergoing MIRPE.

## 2. Materials and Methods

### 2.1. Study Design and Study Population

This retrospective study was conducted at a tertiary university hospital and approved by the Institutional Review Board (KC25RISI0737). The requirement for informed consent was waived due to the retrospective nature of the study. The study was conducted in accordance with the Declaration of Helsinki and reported according to the STROBE guidelines.

Data were collected from the electronic medical records (EMR) of adult patients aged 18 years or older who underwent MIRPE between March 2011 and January 2023. Patients were excluded if they had an American Society of Anesthesiologists (ASA) physical status of III or higher, previous thoracic surgery, including prior MIRPE, preoperative chronic pain or long-term use of analgesic medications, allergy to local anesthetics, or incomplete perioperative data such as pain scores or opioid dosage records.

### 2.2. Surgical Techniques

All operations in this study were performed by a single, experienced surgeon, which ensured technical consistency and minimized heterogeneity within the study cohort. After induction of general anesthesia, the patient was placed in the supine position. Both arms were gently elevated and suspended overhead with arm slings prior to surgical field preparation and draping, thereby securing optimal exposure.

As an initial step, the Easy crane system (Primemed, Seoul, Republic of Korea) was employed to elevate the depressed sternum using either sternal wire sutures or a spiral screw connected to the crane device. This maneuver improved visualization of the operative field and enhanced procedural safety. Following this step, the surgeon determined the appropriate bar length, configuration, hinge points, and incision sites.

Skin incisions, typically 2 cm in length, were made at the midaxillary lines. The number of incisions was determined by the chosen bar configuration. Various bar configurations—including single, multiple parallel, cross, or XI patterns—were applied depending on the deformity, with the ultimate goal of achieving entire chest wall remodeling. Pectoscopic guidance was utilized for safe mediastinal dissection and controlled passage of the pectus bar beneath the elevated sternum. Once the bar was positioned, it was rotated. The resulting chest wall contour was carefully evaluated, and the maneuver was repeated multiple times when necessary to achieve the most satisfactory chest wall contour.

For stabilization, various methods were applied according to the patient’s condition and bar configuration. These included claw fixators, bridge plates, hinge plates, and intercostal sutures, either individually or in combination, to ensure firm anchorage and to prevent bar displacement. Following bar fixation, chest wall contouring was performed using the “magic string” technique and the “flare-buster” method, effectively addressing costal flares and residual protuberances to achieve smooth remodeling of the chest wall.

Finally, portable anteroposterior chest radiography was obtained intraoperatively to confirm accurate bar positioning and to exclude complications such as pneumothorax and hemothorax. Drainage catheters (EZ-VAC silicone type; Gems Korea, Seoul, South Korea) were routinely inserted into the thoracic cavity and removed during the postoperative course as clinically appropriate.

### 2.3. Anesthetic and Analgesic Management

All patients underwent general anesthesia and postoperative pain management according to the institutional protocol for this procedure. General anesthesia was induced with propofol (1.5–2.0 mg/kg) and rocuronium (0.6–1.0 mg/kg), followed by endotracheal intubation. Mechanical ventilation was maintained with a tidal volume of 4–6 mL/kg and an inspired oxygen fraction of 0.4–0.5, keeping end-tidal CO_2_ between 35 and 40 mmHg. Anesthesia was maintained with sevoflurane and continuous remifentanil infusion as part of a balanced anesthesia technique. Sevoflurane was primarily titrated based on a bispectral index of 40–60, and remifentanil was subsequently adjusted to maintain hemodynamic parameters within 20% of baseline. At the end of the procedure, sevoflurane and remifentanil infusion were stopped and residual neuromuscular blockade was reversed with 4 mg/kg sugammadex.

A multimodal analgesic protocol was applied throughout the perioperative periods. After bar placement, intercostal nerve blocks were performed by the surgeon at the fourth to ninth intercostal spaces using 0.5% ropivacaine (2 mL per level, diluted with normal saline). A continuous wound infiltration system (CWIS; ON-Q^®^ Pain Relief System, Halyard, Alpharetta, GA, USA) was placed bilaterally through posterior axillary lines to deliver 0.25% ropivacaine (diluted to a total volume of 300 mL with normal saline) at a rate of 4 mL/h per side. Intravenous patient-controlled analgesia (IV–PCA; AutoMed 3200^®^, ACE Medical Corp., Seoul, Republic of Korea) solution contained fentanyl (20 µg/kg) diluted with normal saline to a total volume of 100 mL. PCA devices were set to deliver a basal infusion of 1 mL/h and a bolus of 1 mL with a lockout interval of 10 min. Both CWIS infusion and IV PCA were initiated immediately upon the patient’s arrival in the post-anesthesia care unit (PACU).

In the PACU, pain intensity was evaluated every 10 min using the visual analog scale (VAS; 0 = no pain, 10 = worst imaginable pain). When the VAS score exceeded 4 despite the use of CWIS and IV PCA, supplemental fentanyl (25–50 µg) or pethidine (12.5–25 mg) was administered intravenously. If pain persisted, intravenous ketorolac (30 mg) was administered as an adjunct rescue analgesic.

During the ward stay, pain scores were assessed every 4 h. All patients routinely received scheduled oral combination analgesics consisting of codeine phosphate 10 mg, ibuprofen 200 mg, and acetaminophen 250 mg three times daily to provide baseline analgesia. Oral medications were not used as rescue analgesics. When breakthrough pain occurred (VAS > 4) despite scheduled oral analgesics, intravenous ketorolac (30 mg) was administered first, followed by intravenous pethidine (25 mg) if pain persisted. If nausea or vomiting occurred, opioid-based PCA was temporarily discontinued and replaced with non-opioid analgesics. The CWIS catheters were routinely removed approximately 24 h postoperatively, at the time of wound drain removal.

### 2.4. Data Collection

Demographic, anatomic, operative, and analgesic data were extracted from electronic medical records. Demographic variables included age, sex, body mass index (BMI), and ASA physical status. Anatomic severity was quantified using the Haller index (HI) and depression index (DI), both calculated from preoperative chest computed tomography (CT) scans. The HI was defined as the ratio of the transverse chest diameter to the anteroposterior distance between the posterior surface of the sternum and the anterior border of the vertebral body [7]. The DI was calculated on the same CT slice as the ratio of the vertical distance from the deepest point of the sternum to a line connecting the most anterior portions of the bilateral ribs, divided by the transverse diameter of the corresponding vertebral body [8]. Operative data included operation time, number and length of inserted bars, and intraoperative fluid balance, calculated as total infused volume minus blood loss. Analgesic-related data included the total opioid dose delivered via PCA and the frequency and dosage of additional rescue analgesics, including both opioid and non-opioid agents.

All opioid consumption (including PCA and rescues) was converted to morphine equivalent dose (MED) normalized by body weight (MED/kg) using standard conversion ratios (10 mg morphine = 100 µg fentanyl = 100 mg pethidine) [9]. Opioid use was analyzed using two complementary measures: (1) interval MED/kg, representing the opioid amount administered within each time window; and (2) cumulative MED/kg, representing the total opioid dose from the end of surgery up to each corresponding time point.

Postoperative pain intensity was evaluated by nurses using the VAS at regular postoperative intervals and documented in the EMR. For analysis, peak VAS values were categorized into three postoperative time windows: 0–6 h, 6–24 h, and 24–48 h after arrival in the PACU. Pain severity was further classified as mild (VAS 0–3), moderate (VAS 4–6), or severe (VAS 7–10).

### 2.5. Outcome

The primary outcome was total opioid consumption during the first 24 postoperative hours. Secondary outcomes included cumulative opioid consumption at 6 and 48 postoperative hours; opioid consumption over 0–6, 6–24, and 24–48 h; rescue requirement across these intervals; temporal changes in pain intensity (peak VAS) and pain-severity distribution within each window.

### 2.6. Statistical Analysis

Continuous variables were tested for normality using the Shapiro–Wilk test and are presented as mean ± standard deviation (SD) or median (interquartile range, IQR), as appropriate. Categorical variables are expressed as counts and percentages. Differences in pain scores and opioid consumption across postoperative time intervals (0–6, 6–24, and 24–48 h) were analyzed using the Friedman test with Bonferroni-adjusted pairwise comparisons.

To identify factors independently associated with postoperative opioid consumption, multivariable linear regression analysis was performed. Covariates were selected a priori based on previously published data and clinical relevance, including age, sex, BMI, HI, DI, operation time, and the number and length of inserted bars. Ordinal logistic regression was applied to explore associations between the same covariates and pain severity (mild, moderate, or severe). Statistical significance was defined as a two-sided *p* < 0.05. All analyses were performed using IBM SPSS Statistics for Windows, version 25.0 (IBM Corp., Armonk, NY, USA).

## 3. Results

### 3.1. Baseline Characteristics of the Study Population and Intraoperative Variables

Data were retrospectively collected from patients who underwent MIRPE between March 2011 and January 2023. Patients with missing or incomplete data were excluded, resulting in a total of 382 patients included in the final analysis. The baseline demographic and surgical characteristics of the study population are summarized in Table 1. The mean age was 22.5 ± 5.0 years, and 80.6% of the patients were male. The mean BMI was 25.9 kg/m^2^. The mean HI and DI were 4.9 ± 3.2 and 1.9 ± 1.0, respectively. Most patients underwent bar insertion with fewer than two bars and bar lengths shorter than 14 inches. No major intraoperative complications, such as bleeding or pneumothorax, were observed.

### 3.2. Postoperative Opioid Requirements and Pain Intensity

Postoperative opioid consumption and pain intensity exhibited significant time-dependent variations during the first 48 h after surgery (Table 2). Opioid use increased over time, with the largest amount administered within the first 24 h postoperatively (*p* < 0.001). Pain intensity, as indicated by the peak VAS score, was highest during the initial 0–6 h and decreased markedly during the subsequent 6–24 h (*p* < 0.001). A mild but statistically significant increase in pain intensity was again observed during a 24–48 h period (*p* < 0.001).

**Figure 1:**
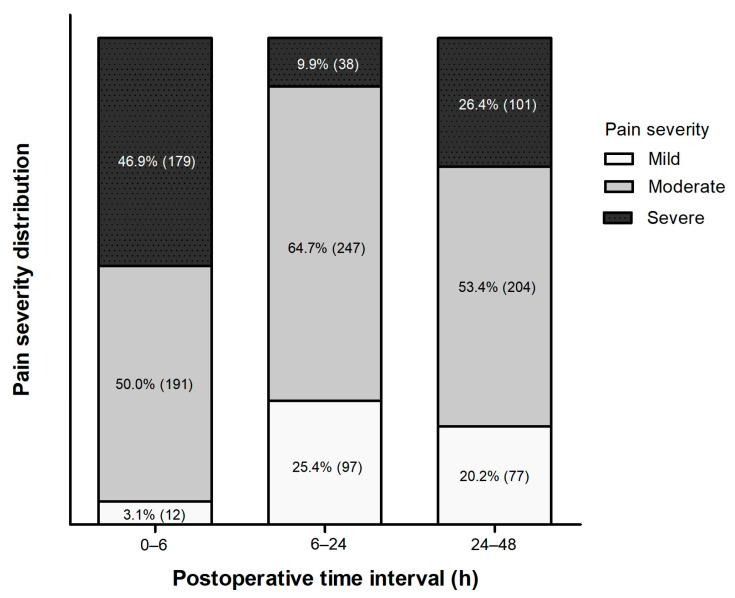
Temporal changes in the proportion of patients by pain severity during the first 48 h after adult MIRPE. Values are expressed as the proportion of patients (the number of patients). Mild = VAS 0–3; Moderate = VAS 4–6; Severe = VAS 7–10.

The proportion of patients experiencing severe pain (VAS score ≥ 7) was highest during the initial 0–6 h (*n* = 179, 46.9%), then decreased markedly during 6–24 h (*n* = 38, 9.9%). However, it increased again during 24–48 h postoperatively (*n* = 101, 26.4%) (Figure 1).

**Figure 2:**
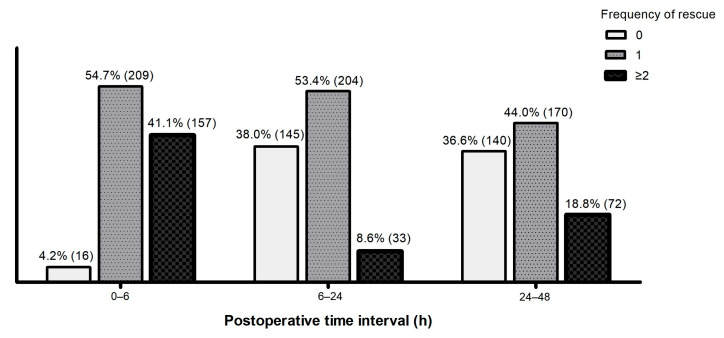
Frequency of rescue analgesic administration after adult MIRPE. Values are expressed as the proportion of patients (the number of patients).

Rescue analgesics were administered to nearly all patients across the three postoperative intervals (0–6, 6–24, and 24–48 h). The proportion of patients who required two or more doses of PRN medication increased during 24–48 h compared with 6–24 h (Figure 2).

### 3.3. Predictors of Postoperative Opioid Consumption and Pain Severity

Multivariable linear regression analyses for postoperative opioid consumption at 6, 24, and 48 h are summarized in Table 3. During the first 6 h, insertion of more than three bars and higher BMI were independently associated with greater opioid requirements (*p* < 0.001). At 24 h—the primary analysis time point—male sex, longer operation time, and higher BMI were significant predictors of increased opioid use (*p* < 0.01). These associations persisted up to 48 h postoperatively, with male sex and operation time demonstrating the strongest and most consistent influence on cumulative opioid consumption (Supplementary Tables S1–S3).

Ordinal logistic regression analyses for postoperative pain severity are summarized in Table 4. During the first 6 h, patients with insertion of more than three bars reported significantly higher pain severity (*p* < 0.001). From 6 to 24 h, male sex and longer operation time were independently associated with more severe pain (*p* < 0.01). These associations remained significant at 48 h, indicating that male patients and those undergoing longer procedures consistently experienced greater postoperative pain. Neither the Haller index nor the depression index was significantly associated with pain severity at any time point.

## 4. Discussion

This study aimed to identify perioperative factors affecting postoperative opioid use and pain intensity during the early postoperative phase in adult patients undergoing MIRPE. Among the variables examined, male sex, higher BMI, longer operation time, and a greater number of implanted bars were significantly associated with increased opioid requirements and pain severity after surgery. In contrast, anatomical severity indices such as the HI and the DI showed no significant correlation with either opioid use or pain scores. These findings suggest that postoperative discomfort during the first 48 h after MIRPE in adults is primarily influenced by patient- and surgery-related factors rather than the severity of anatomical deformity.

Compared to earlier reports, our findings were partially consistent but also showed differences, particularly regarding gender-related variations in early postoperative pain. Most previous studies, mostly involving pediatric or mixed-age groups, have yielded inconsistent results: some indicated that female patients experienced higher pain scores and complication rates [10,11], whereas others reported the opposite [12,13]. In our adult group, male patients reported greater pain intensity and required higher opioid doses during the early postoperative period after MIRPE. Anatomical and biomechanical features of the adult thorax may explain these results. Stiffness of the costal cartilage is greater in males than in females [14], leading to greater mechanical stress on the intercostal nerves and surrounding muscles during bar rotation and fixation [15]. Additionally, males typically have thicker chest walls and more muscle mass, which can increase intercostal tension and decrease tissue flexibility [16,17]. Overall, these anatomical factors likely contribute to increased nociceptive activation, resulting in more postoperative discomfort and higher opioid needs in male patients.

The relationship between the number of inserted bars and postoperative pain has also varied across studies. One biomechanical analysis suggested that double-bar placement reduced postoperative pain during the first two postoperative days by more evenly distributing chest wall stress [18]. However, our findings showed that patients requiring three or more bars experienced greater pain in the early postoperative period. This difference may reflect the broader correction range and greater mechanical force applied across each intercostal space, rather than the number of bars itself [19,20]. Similarly, longer operation times often indicated more extensive or complex corrections, during which multiple bars were adjusted or repositioned to achieve proper correction, thereby increasing mechanical stress on the chest wall.

The absence of a significant link between deformity indices (HI and DI) and postoperative pain in our adult group differs from previous pediatric studies [6,13]. These indices measure geometric deformity but not structural rigidity, which increases significantly with age as the costal cartilage ossifies [21]. In pediatric patients, more severe deformities may cause more pain due to the elastic recoil of the flexible chest wall [22]. In contrast, in adults, ossified ribs and reduced thoracic compliance are the main factors influencing pain, decreasing the impact of geometric deformity on pain perception. This interpretation is supported by biomechanical evidence showing that the force required to achieve a similar level of sternal elevation grows exponentially with increasing thoracic stiffness [15,23].

Although BMI was not linked to differences in reported pain intensity, patients with higher BMI consistently needed higher opioid doses across all postoperative periods. This suggests that opioid requirements do not necessarily match subjective pain perception and may be influenced by multiple interacting factors. Individuals with higher body mass may have altered pharmacokinetics of lipophilic opioids such as fentanyl, including increased distribution into adipose tissue and decreased effective plasma concentrations [24]. Therefore, even at similar pain levels, patients with higher BMI may require larger doses of opioids to achieve equivalent analgesic effects.

An additional notable observation was the biphasic postoperative pain pattern, characterized by an initial peak within 6 h, a transient decline by 24 h, and a secondary rise at 24–48 h. In most surgical populations, postoperative pain generally peaks within the first 24–36 h and gradually decreases thereafter [25]. However, in our cohort, pain did not continue to decrease after 24 h but instead increased again, accompanied by a parallel rise in rescue analgesics use. While a causal relationship cannot be definitively established, the timing of this secondary increase coincided with the routine removal of the CWIS, suggesting that termination of local anesthetic delivery may expose residual inflammation and peripheral sensitization. Similar rebound phenomena have been reported following discontinuation of epidural or local infiltration in thoracic and upper abdominal surgeries, likely mediated by reactivation of inflammatory pathways as local blockade subsides [26,27].

From a clinical perspective, these findings highlight the importance of a risk-based, time-sensitive analgesic approach during the immediate postoperative period following MIRPE. Male patients, those with higher BMI, and individuals undergoing longer procedures should be identified before surgery as high-risk groups for postoperative pain and managed with tailored multimodal strategies. In such cases, extending local or regional analgesic coverage beyond 24 h—by using prolonged CWIS infusions or scheduled regional blocks—may mitigate late pain rebound. Additionally, opioid doses based on lean or adjusted body weight rather than total body weight could enhance pharmacologic accuracy in heavier adults [28]. Lastly, patients requiring three or more bars need intensified pain management and close monitoring during the first six hours after surgery to avoid breakthrough pain. Implementing individualized multimodal interventions during the early postoperative phase may improve pain control after MIRPE.

This study has several limitations. First, its retrospective design may have introduced selection and information bias. Second, as a single-center study, the findings may not be generalizable to institutions with different perioperative practices or patient populations. Third, pain assessment relied solely on the VAS measured at predefined time intervals, without incorporating multidimensional outcomes such as patient satisfaction or functional recovery. Finally, the follow-up period was limited to 48 h; therefore, the identified risk factors should not be extrapolated to the entire postoperative course or long-term pain outcomes. Nevertheless, the relatively large and homogeneous adult cohort, combined with standardized surgical techniques and consistent pain documentation, supports the internal validity of the findings and provides clinically meaningful insights into early postoperative pain management after MIRPE. Future prospective studies are warranted to validate these findings with an extended follow-up period and to explore optimized CWIS duration, adjunctive regional techniques, and individualized analgesic protocols for adult MIRPE.

## 5. Conclusions

In conclusion, early postoperative pain and opioid requirements after adult MIRPE during the first 48 h after surgery were associated with male sex, higher BMI, longer operation duration, and multiple-bar insertion, rather than with deformity severity. Pain showed a secondary increase around 24–48 h, coinciding with CWIS removal. These findings highlight the need for personalized and time-specific multimodal analgesic strategies to provide adequate pain control.

## Figures and Tables

**Table 1:** Baseline characteristics of the study population.

Variables	All Patients (*n* = 382)
Age (year)	22.5 ± 5.0
Gender	
Male	308 (80.6%)
Female	74 (19.4%)
Height (cm)	173.6 ± 10.5
Weight (kg)	61.0 ± 9.3
BMI (kg/m^2^)	25.9 ± 3.4
Haller index	4.9 ± 3.2
Depression index	1.9 ± 1.0
Bar number (≤2)	277 (72.5%)
Bar length (≤14 inch)	244 (63.9%)
Operation time (min)	134.7 ± 33.0
Fluid balance (mL)	396.5 ± 251.3

Values are expressed as numbers (proportion) or mean ± standard deviation. (BMI = body mass index; Fluid balance = infused fluid volume-blood loss).

**Table 2:** Summary of postoperative opioid consumption and pain scores at 6, 24, and 48 h.

Time Interval	Interval MED/kg	Cumulative MED/kg	Peak VAS
0–6 h	0.14 ± 0.19	0.14 ± 0.19	6.2 ± 1.1
6–24 h	1.04 ± 0.58	1.17 ± 0.65	4.5 ± 1.6
24–48 h	1.32 ± 0.70	2.49 ± 1.31	5.3 ± 1.7

Values are expressed as mean ± standard deviation. (MED = morphine equivalent dose; VAS = visual analog scale).

**Table 3:** Multivariable linear regression analysis for factors associated with postoperative opioid consumption at (**a**) 6 h, (**b**) 24 h and (**c**) 48 h after adult MIRPE.

(a) 6 h cMED/kg					
	B	β	t	*p*-value	VIF
Bar ≥ 3	0.145	0.343	6.430	<0.001	1.06
BMI	0.000	0.098	2.004	0.046	1.01
R^2^ = 0.238, adj R^2^ = 0.220, F = 7.47, *p* < 0.001, Durbin-Watson = 1.826					
**(b) 24 h cMED/kg**					
	**B**	**β**	**t**	***p*** **-value**	**VIF**
Male	0.356	0.217	3.992	<0.001	1.22
Operation time	0.003	0.167	2.875	0.004	1.52
BMI	0.001	0.106	2.124	0.034	1.2
R^2^ = 0.297, adj R^2^ = 0.277, F = 4.98, *p* < 0.001, Durbin-Watson = 2.074					
**(c) 48 h cMED/kg**					
	**B**	**β**	**t**	***p*** **-value**	**VIF**
Male	0.612	0.185	3.386	0.001	1.07
Operation time	0.007	0.167	2.854	0.005	1.52
BMI	0.001	0.114	2.278	0.023	1.06
R^2^ = 0.287, adj R^2^ = 0.267, F = 4.42, *p* < 0.001, Durbin-Watson = 2.084					

BMI = body mass index; cMED = cumulative morphine equivalent dose.

**Table 4:** Ordinal logistic regression analysis of predictors of postoperative pain severity at (**a**) 6 h, (**b**) 24 h and (**c**) 48 h after adult MIRPE.

(a) 6 h VAS Severity			
	OR	95% CI	*p*-value
Bar ≥ 3	2.2	2.01–2.43	<0.001
**(b) 24 h VAS severity**			
	**OR**	**95% CI**	***p*** **-value**
Male	3.45	1.94–6.13	<0.001
Operation time	2.02	2.01–2.03	<0.001
**(c) 48 h VAS severity**			
	**OR**	**95% CI**	***p*** **-value**
Male	3.76	2.10–6.74	0.003
Operation time	1.95	1.12–3.39	0.016

OR = odds ratio; CI = confidence interval; VAS = visual analog scale.

## Data Availability

The data generated in this study can be shared after a reasonable request to the corresponding author.

## Supplementary Materials

The following supporting information can be downloaded at: https://www.mdpi.com/article/10.3390/jcm15031023/s1, Table S1. Multivariable linear regression analysis for postoperative opioid consumption at 6 h after adult MIRPE. Table S2. Multivariable linear regression analysis for postoperative opioid consumption at 24 h after adult MIRPE; Table S3. Multivariable linear regression analysis for postoperative opioid consumption at 48 h after adult MIRPE.

## Abbreviations

The following abbreviations are used in this manuscript:

ASA	American society of anesthesiologists
BMI	Body mass index
CWIS	Continuous wound infiltration system
DI	Depression index
EMR	Electronic medical records
HI	Haller index
MED	Morphine equivalent dose
MIRPE	Minimally invasive repair of pectus excavatum
NSAID	Nonsteroidal anti-inflammatory drug
PACU	Post-anesthesia care unit
PCA	Patient-controlled analgesia
PE	Pectus excavatum
VAS	Visual analog scale
