# Supplementary material for: Analysis of Factors Affecting Postoperative Opioid Requirement in Adult Patients Undergoing Minimally Invasive Repair of Pectus Excavatum
Source: J Clin Med. 2026 Jan 27;15(3):1023. doi: 10.3390/jcm15031023 (PMC12897850; doi:10.3390/jcm15031023)
Supplement: Supplementary file 1 [file jcm-15-01023-s001.zip › jcm-4051778-supplementary.pdf]

Supplementary tables present all multivariable linear regression analyses performed to identify factors associated with postoperative opioid consumption. All models used identical covariates including age, gender, body mass index (BMI), Haller index (HI), depression index (DI), operation time, and the number and length of inserted bars.

Supplementary Table S1. Multivariable linear regression analysis for postoperative opioid consumption at 6 hours after adult MIRPE

Variable	B	95% CI	β	*p*- value
Age (yr)	0.001	-0.001 to 0.003	0.054	0.242
Male	0.001	-0.027 to 0.024	0.005	0.917
BMI	0.007	0.003 to 0.012	0.143	0.002
HI	0.005	-0.019 to 0.010	0.029	0.528
DI	0.042	-0.186 to 0.102	0.026	0.566
Operation time (min)	0.000	-0.001 to 0.000	0.010	0.823
Number of bars ≥ 3	0.131	0.103 to 0.158	0.430	<0.001
Length of bar ≥ 15 inch	0.021	-0.003 to 0.044	0.079	0.085

Supplementary Table S2. Multivariable linear regression analysis for postoperative opioid consumption at 24 hours after adult MIRPE

Variable	B	95% CI	β	*p*- value
Age (yr)	-0.004	-0.009 to 0.000	-0.081	0.053
Male	0.364	0.309 to 0.420	0.536	<0.001
BMI	0.011	0.001 to 0.021	0.089	0.034
HI	-0.025	-0.057 to 0.007	-0.064	0.126
DI	-0.114	-0.429 to 0.201	-0.030	0.480
Operation time (min)	0.004	0.002 to 0.005	0.248	<0.001
Number of bars ≥ 3	0.002	-0.058 to 0.061	0.003	0.951
Length of bar ≥ 15 inch	0.026	-0.025 to 0.077	0.042	0.325

Supplementary Table S3. Multivariable linear regression analysis for postoperative opioid consumption at 48 hours after adult MIRPE

**Variable**	**B**	**95% CI**	**β**	***p*- value**
Age (yr)	0.003	-0.007 to 0.013	0.026	0.547
Male	0.636	0.515 to 0.757	0.450	<0.001
BMI	0.044	0.022 to 0.065	0.171	<0.001
HI	0.044	-0.025 to 0.113	0.054	0.215
DI	0.146	-0.536 to 0.829	0.018	0.675
Operation time (min)	0.008	0.005 to 0.010	0.256	<0.001
Number of bars ≥ 3	0.111	-0.019 to 0.240	0.073	0.094
Length of bar ≥ 15 inch	0.027	-0.084 to 0.137	0.021	0.639
